# A MADS-Box Gene *CiMADS43* Is Involved in Citrus Flowering and Leaf Development through Interaction with CiAGL9

**DOI:** 10.3390/ijms22105205

**Published:** 2021-05-14

**Authors:** Li-Xia Ye, Jin-Xia Zhang, Xiao-Jin Hou, Mei-Qi Qiu, Wen-Feng Wang, Jin-Xin Zhang, Chun-Gen Hu, Jin-Zhi Zhang

**Affiliations:** Key Laboratory of Horticultural Plant Biology (Ministry of Education), College of Horticulture and Forestry Science, Huazhong Agricultural University, Wuhan 430070, China; yelixia@webmail.hzau.edu.cn (L.-X.Y.); zhjinxia@webmail.hzau.edu.cn (J.-X.Z.); houxiaojin1988@126.com (X.-J.H.); meiqi0527@webmail.hzau.edu.cn (M.-Q.Q.); wangwenfeng@webmail.hzau.edu.cn (W.-F.W.); zhang-jinxin1@webmail.hzau.edu.cn (J.-X.Z.); chungen@mail.hzau.edu.cn (C.-G.H.)

**Keywords:** citrus, *CiMADS43*, *CiAGL9*, flowering, leaf

## Abstract

MADS-box genes are involved in various developmental processes including vegetative development, flower architecture, flowering, pollen formation, seed and fruit development. However, the function of most MADS-box genes and their regulation mechanism are still unclear in woody plants compared with model plants. In this study, a MADS-box gene (*CiMADS43*) was identified in citrus. Phylogenetic and sequence analysis showed that *CiMADS43* is a GOA-like Bsister MADS-box gene. It was localized in the nucleus and as a transcriptional activator. Overexpression of *CiMADS43* promoted early flowering and leaves curling in transgenic *Arabidopsis*. Besides, overexpression or knockout of *CiMADS43* also showed leaf curl phenotype in citrus similar to that of *CiMADS43* overexpressed in *Arabidopsis*. Protein–protein interaction found that a SEPALLATA (SEP)-like protein (CiAGL9) interacted with CiMADS43 protein. Interestingly, *CiAGL9* also can bind to the *CiMADS43* promoter and promote its transcription. Expression analysis also showed that these two genes were closely related to seasonal flowering and the development of the leaf in citrus. Our findings revealed the multifunctional roles of *CiMADS43* in the vegetative and reproductive development of citrus. These results will facilitate our understanding of the evolution and molecular mechanisms of MADS-box genes in citrus.

## 1. Introduction

The MADS-box gene family is involved in a variety of biological processes such as vegetative development, flowering, seed and fruit development [1]. MADS-box genes can be divided into two types depending on the characteristics of the consensus amino acid sequence in the MADS-box domain [2]. Type I, the M-type, contains the conserved M domain with a large variable region at the C-terminus and classifies into three subclasses (Mα, Mβ, and Mγ) [3]. Type II, known as MIKC domain, is composed of MADS domain, I domain, K-domain and C region from *N*- to *C*-termini [4]. In model plants, MADS-box genes have been reported as floral organ identity genes which can be summarized as the ABC model and then extended to the ABCDE model [5]. In this model, almost all genes except *APETALA2* (*AP2*) belong to the MIKC-type MADS-box subfamily, indicating that MIKC-type MADS-box genes play a key role during flower development [6]. In the past few decades, a large number of MADS-box genes related to flower development are also identified in woody plants including citrus [7,8,9]. However, the specific regulation mechanism is still unclear compared with model plants.

Besides flower development, several MIKC-type genes have been also confirmed to be involved in plant flowering [10,11,12]. For example, *FLOWERING LOCUS C* (*FLC*) encodes a specific MADS-box protein and as a typical flowering suppressor [10,11,12]. Citrus *FLC* homolog is conserved with model plants, but it regulates the phase changes of citrus through alternative splicing [13,14]. *AGAMOUSLIKE16* (*AGL16*) is a target of miR824, which inhibits plant flowering [15,16]. *SUPPRESSOR OF OVEREXPRESSION OF CONSTANS1* (*SOC1*) acts as a direct target gene for *CONSTANS* (*CO*) to integrating vernalization and gibberellin pathways [17,18]. *AtAGL12* is an upstream regulator of *SOC1*, *FLOWERING LOCUS T* (*FT*), and *LEAFY* (*LFY*), and as a flowering-promoting factor [19]. *AFFECTING FLOWERING* (*MAF1/FLM*), *AGL12*, *AGL15*, *AGL18*, and *AGL24* are involved in flowering regulation as activators [19,20,21,22,23]. Moreover, earlier studies reported that some MADS-box genes are involved in regulating the development of stem apical meristem (SAM). For example, *PkMADS1* is involved in maintaining the interaction between leaf primordium and SAM in *Paulownia kawakamii* [24]. Another MADS-box gene (*POTM1*) from potato specifically accumulates in vegetative growth meristem and regulates the formation of axillary buds [25]. In sugarcane, *SOC1* and *SHORT VEGETATIVE PHASE* (*SVP*) mainly regulate vegetative growth and are detected in leaves, roots, and stems [26]. Overexpression *SHATTERPROOF 2* (*IiSHP2*) from *Isatis indigotica* produced shortened sepals that cannot completely encapsulate the flower buds in transgenic *Arabidopsis* [27]. These previous results suggested that some MADS-box genes have diverse roles in both vegetative and reproductive development [26,28,29]. However, their regulation mechanism need further analysis.

The regulation mechanism of many MADS-box genes is established by interacting with other MADS-box proteins. For example, OsMADS29 can form heterodimers with its closely related paralog OsMADS31 in rice and regulate seed development [30,31]. In *Arabidopsis*, *AGL24* and *SVP* involved in bud development and floral transition through interacting with other proteins at different stages of development [20]. At the vegetative stage, *SVP* and *FLC* down-regulates *SOC1* and *FT* by forming a complex [32,33]. At the early stages of flower development, *SVP* and *AGL24* act redundantly and interact with *APETALA1* (*AP1*) to maintain floral meristem identity [34,35,36]. In citrus, the functions and regulatory mechanisms of *SVP* and *AGL24* are also conserved, and they cooperate with AP1 protein to control flowering and flower development [9,37]. In addition, *SVP* was also involved in dormancy, drought stress, and flower color regulation in other woody plants [38,39,40,41].

It is noteworthy that a new group MIKC MADS-box genes are close relatives with the B-type genes and are referred to as Bsister genes [42]. Bsister proteins have been found in both Gymnosperm and Angiosperm species [43,44,45,46]. Bsister genes have been reported to be critical for ovule and seed development in *Arabidopsis* and rice [42,47,48,49]. *ARABIDOPSISBSISTER* (*ABS*) of *Arabidopsis* is the first Bsister gene and has been shown to regulate proper endothelial differentiation and proanthocyanidin (PA) accumulation in the seed coat [29,47,48,50]. The other Bsister gene *GORDITA* (*GOA*) of *Arabidopsis* has a new and non-redundant function for *ABS* in regulating ovule coat development and fruit longitudinal growth [51]. Overexpression of *AtGOA* results in early flowering, curly leaf and extremely dwarfing [28,51]. In *Brassica napus*, the RNAi plants of *ABS* homolog *TRANSPARENT TESTA 16* (*TT16*) have seeds with deformed or completely lacking embryo [49]. Similar to eudicots, the Bsister genes of monocots are also involved in seed development. *OsMADS29* regulates the expression of genes which is necessary for programmed cell death (PCD) in the nuclear region of the developing seeds cell [45,52]. Another mutant allele with loss function of *OsMADS29* exhibits a sterile phenotype due to lack of embryo and endosperm development [53]. Unfortunately, although information is beginning to accumulate with regard to the ability of Bsister genes to regulate plant development in model plants or annual crops, its precise regulatory function remains largely unknown in woody plants such as citrus.

In a previous study, a total of 52 MADS-box genes were identified in sweet orange using bioinformatics analysis [7], only two Bsister genes were found in citrus. Sequence alignment and phylogenetic analysis demonstrated that *CiMADS43*, a homologous of *COA* in *Arabidopsis*, is a typical Bsister subfamily MIKC type MADS-box gene. *CiMADS43* is a typical transcriptional activator and mainly expresses in flower, fruit, and SAM. We speculated that *CiMADS43* may also play a key role in the development of citrus, similar to model plants [28,54]. Therefore, it was selected for further analysis in citrus. In this study, overexpression of *CiMADS43* resulted in early flowering and curled leaves in *Arabidopsis*. Furthermore, overexpression or knockout of *CiMADS43* in citrus also showed the leaf curl phenotype similar to that of transgenic *Arabidopsis.* CiAGL9 can interact with CiMADS43 protein and also bind to the *CiMADS43* promoter. Overall, these results suggested that *CiMADS43* regulated flowering and leaf development through interaction with CiAGL9. Our study will better our understanding of the complex regulation of Bsister gene in perennial woody.

## 2. Results

### 2.1. Isolation and Characterization of the CiMADS43 Gene

**Figure 1:**
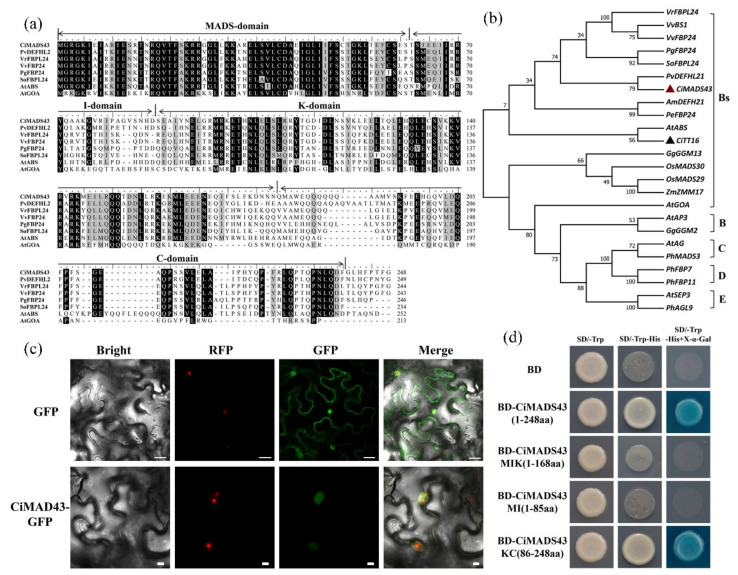
Characterization of the *CiMADS43*. (**a**) Multiple sequence alignment of CiMADS43 protein and its homolog proteins. CiMADS43, *Citrus sinensis* (XP_006468501.1). PvDEFHL21, *Pistacia vera* (XP_031278613.1). VrFBPL24, *Vitis riparia* (XP_034698718.1). VvFBP24, *Vitis vinifera* (XP_010648959.1). PgFBP24, *Punica granatum* (XP_031400121.1). SoFBPL24, *Syzygium oleosum* (XP_030467505.1). AtABS, *Arabidopsis thaliana* (NP_001330404.1). AtGOA, *Arabidopsis thaliana* (NP_174399.2). (**b**) Phylogenetic analysis of MADS-box proteins from citrus and other plants. The gene ID of all MADS-box proteins were listed in Table S1. The red triangle indicates the citrus *GORDITA* (GOA)-like Bsister gene and the black triangle indicates the citrus ABS-like Bsister gene. (**c**) Subcellular localization of CiMADS43 in tobacco leaves. Green fluorescent protein (GFP) fused to the *C*-terminal region of *CiMADS43*, and the fusion protein was driven by 35S promoter. Red fluorescent protein (RFP) label was used as a nuclear marker driven by 35S promoter, and 35S::GFP was used as positive control. Scale bar = 5 0 µm. (**d**) Analysis of CiMADS43 transcription activation. CiMADS43 represents a full-length protein, and CiMADS43MIK was a truncated protein that the *C*-terminal region was removed, containing M domain, I domain and K domain. CiMADS43MI indicates the *N*-terminal region of CiMADS43 protein including M domain and I domain, while CiMADS43KC indicates the *C*-terminus of CiMADS43 protein including K domain and C domain.

In a previous study, the citrus MADS-box gene family was comprehensively identified and characterized [7]. Among these MADS-box genes, a typical MIKC type MADS-box gene (*CiMADS43*) was identified in sweet orange. The coding region sequence of *CiMADS43* was 747 bp, and encoded a protein of 248 amino acids. CiMADS43 contained four conserved MIKC domains. The highly conserved MADS (M) domain was located at the *N*-terminus, which binds to DNA and exists as hetero and homo-dimers. The intervening (I) domain was variable. The keratin-like (K) domain is a possible coiled-coil structure that may play a role in multimer formation. While the C domain at the *C*-terminus has the most species diversity (Figure 1a). Phylogenetic analysis indicated that CiMADS43 belonged to the Bsister subfamily of MADS-box proteins (Figure 1b).

To analyze the subcellular localization of *CiMADS43*, the *35S::CiMADS43-GFP* (Green fluorescent protein) fusion vector and *35S::GFP* empty vector (control) were transiently expressed in epidermal cells of tobacco leaves. Red fluorescent protein (RFP) labeled was used as a nuclear marker to indicate the location of the nucleus [55]. The green fluorescence of the *CiMADS43-GFP* fusion proteins was observed in the nucleus, whereas the signals of the control were distributed on the whole cell (Figure 1c). These results demonstrated that CiMADS43 was located in the nucleus. In addition, transcription activation assay was performed in yeast by fusing the full-length or truncated CiMADS43 proteins with the yeast GAL4-BD. CiMADS43 was a full-length protein, while CiMADS43MIK was a truncated protein that the *C*-terminal region was removed, containing M domain, I domain and K domain. CiMADS43MI was the *N*-terminal region of CiMADS43 protein including M domain and I domain, while CiMADS43KC was the *C*-terminus of CiMADS43 protein including K domain and C domain. Yeast cells with BD-CiMADS43 or BD-CiMADS43KC grew well on yeast synthetic drop-out medium SD-Trp/-His plates and catalyzed 5-bromo-4-chloro-3-indolyl-a-D-galactopyranoside (X-a-Gal), while yeast cells transformed with BD-CiMADS43MIK or BD-CiMADS43MI could not grow normally on SD-Trp/-His plates and could not catalyze the degradation of X-a-Gal (Figure 1d). These results indicated that *CiMADS43* has transcription activity and the *C*-terminal region might possess the transcription activation property.

### 2.2. Functional Analysis of CiMADS43 in Arabidopsis

**Figure 2:**
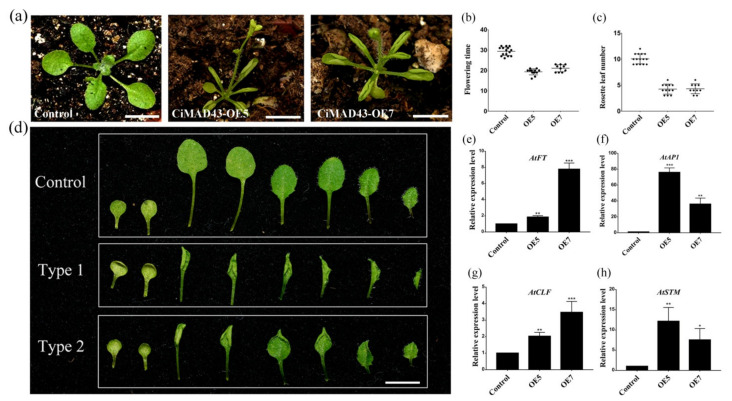
Ectopic expression of *CiMADS43* caused early flowering and leaf curling in transgenic *Arabidopsis*. (**a**) *CiMADS43* transgenic *Arabidopsis* significantly earlier flowering than the control. Two transgenic lines (CiMADS43-OE5 and CiMADS43-OE7) were selected as representatives of 12 transgenic lines for evaluating *MADS43* function. (**b**) Statistical analysis of the flowering days of the *CiMADS43* transgenic *Arabidopsis*. (**c**) Statistical analysis of the number of rosette leaves when the *CiMADS43* transgenic *Arabidopsis* and the control begin to flowering. (**d**) Two types of curling rosette leaf from *CiMADS43* transgenic *Arabidopsis*. Type 1: all rosette leaves except cotyledons severely curly. Type 2: only some rosette leaves slightly curly. Arranged from left to right according to different stages of leaf development, with the cotyledons on the far left. (**e**,**f**) Quantitative real-time polymerase chain reaction (qRT-PCR) analysis of *Arabidopsis* flowering-related genes (*AtFT* and *AtAP1*) in the *CiMADS43* transgenic *Arabidopsis.* (**g**,**h**) qRT-PCR analysis of *Arabidopsis* leaf development related genes (*CLF* and *STM*). Scale bar = 0.5 cm. Statistically significant is marked with asterisk(s) (* *p* < 0.05; ** *p* < 0.01; and *** *p* < 0.001; Student’s *t*-test).

To investigate the function of *CiMADS43*, *CiMADS43* was overexpressed in wild type *Arabidopsis*. A total of 12 transgenic lines were obtained. Compared with the control, these transgenic plants flowered significantly earlier (Figure 2a). Two transgenic lines was selected as representatives of 12 transgenic lines for evaluating *CiMADS43* function. The results showed that the average flowering time of transgenic plants is 16 to 23 days, while the average flowering time of the control is 29.4 days (Figure 2b). In transgenic plants, the average number of rosette leaves at flowering is 3 to 6, while the average number of the control rosette leaves is 10.1, which is significantly less than the control (Figure 2c). In addition, the rosette leaves of these transgenic lines showed different degrees of curl, and the cotyledons were normal (Figure S1). The phenotype of transgenic plants were divided into two types according to the degree of rosette leaves curl (Type 1 and Type 2). Type 1: all rosette leaves except cotyledons severely curly. Type 2: only some rosette leaves slightly curly (Figure 2d).

To investigate the mechanism of *CiMADS43* regulating flowering and leaf development, the expression of some *Arabidopsis* endogenous genes related flowering and leaf development was investigated in transgenic plants by quantitative real-time polymerase chain reaction (qRT-PCR). *FT* and *AP1* are the key genes of plant flowering [56,57], and their expression were significantly upregulated in transgenic *Arabidopsis* compared with the control (Figure 2e,f). In some previous reports, *CURLY LEAF* (*CLF*, AT2G23380.1), *SHOOTMERISTEMLESS* (*STM*, AT1G62360.1) participate in leaf development [58]. Therefore, their expression levels were analyzed in *CiMADS43* transgenic plants. These two genes were significantly up-regulated (Figure 2g,h). These results indicated that *CiMADS43* may be involved in flowering and leaf development of citrus.

### 2.3. Overexpression and Knockout of CiMADS43 in Citrus

**Figure 3:**
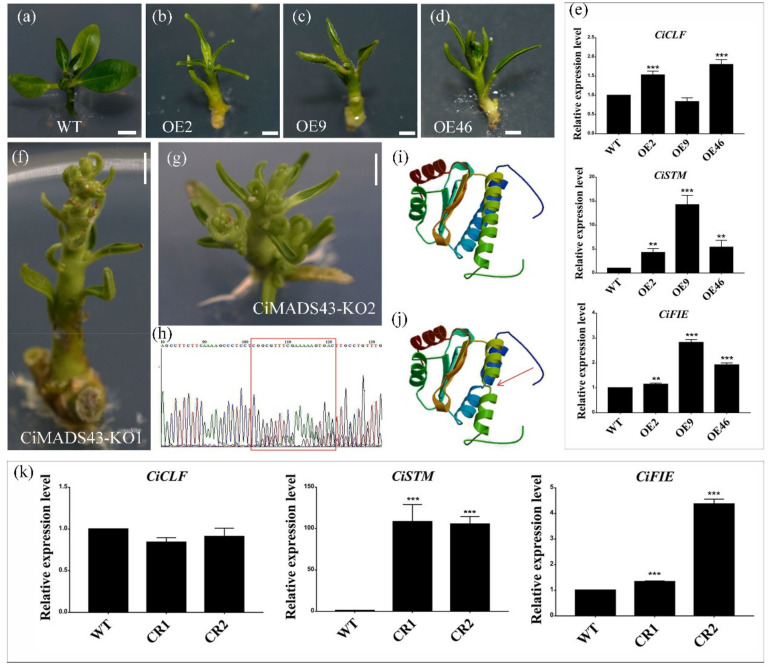
Overexpression and knockout of *CiMADS43* in lemon. (**a**) Wild type lemon. (**b**–**d**) The phenotype of three overexpressed citrus transgenic lines with *CiMADS43*. (**e**) qRT-PCR analysis of citrus leaf development related genes (*CLF*, *STM*, and *FIE*) in *CiMADS43* overexpression lines. (**f**,**g**) Phenotypic analysis of *CiMADS43* knockout lines by CRISPR/Cas9 system. (**h**) DNA sequencing analysis of gRNA target in *CiMADS43* knockout lines. The red box indicates the position of the gRNA target. Double peaks appears due to the deletion of three nucleotides adjacent to the PAM (Protospacer Adjacent Motif) region. (**i**,**j**) The three-dimensional structure of CiMADS43 protein (**i**) and the residual protein which knocked out three bases by CRISPR/Cas9 system (**g**). The red arrow indicates the location of the change. (**k**) qRT-PCR analysis of citrus leaf development related genes (*CLF*, *STM*, and *FIE*) in *CiMADS43* knockout lines. Scale bar = 0.2 cm. Statistically significant is marked with asterisk(s) (** *p* < 0.01 and *** *p* < 0.001, Student’s *t*-test).

To further confirm the function of *CiMADS43* in citrus, it was also overexpressed in citrus. The transformation of sweet orange is very difficult because of the limitation of explants, so we use lemon for transformation. Lemon and sweet orange belong to different citrus species. A total of 9 transgenic citrus lines were obtained. Compared with the control, *CiMADS43* showed a higher expression level in each transgenic citrus line (Figure S2). Phenotypic analysis found that most of the transgenic citrus plants also showed a phenotype of abnormal leaf development (Figure 3a–d). These results further confirm that *CiMADS43* was involved in leaf development, and also indicate that the phenotype of transgenic *Arabidopsis* was reliable. However, early flowering of transgenic citrus cannot be observed because of the long juvenile stage in citrus. Compared with the control, the leaf development related genes *CLF* (Ciclev10024826m), *STM* (Ciclev10015581m) and *FERTILIZATION-INDEPENDENT ENDOSPERM* (*FIE*, Ciclev10008697m) were upregulated in the transgenic plants (Figure 3e).

Meanwhile, the Clustered Regularly Interspaced Short Palindromic Repeats (CRISPR) system was also employed in lemon to verify the function of *CiMADS43*. A total of four knockout transgenic citrus lines were obtained, and sequencing analysis revealed that *CiMADS43* was edited in two knockout transgenic citrus lines (Figure 3f,g). DNA sequencing indicated that three nucleotides adjacent to the PAM (Protospacer Adjacent Motif) region was deleted (Figure 3h and Figure S3a). Protein sequence analysis found that one amino acid in the M domain of CiMADS43 protein was deleted (Figure S3b). Its three-dimensional structure was significantly changed by the protein structure prediction (Figure 3i,j). Interestingly, the leaves of *CiMADS43* CRISPR lines present a phenotype similar to its overexpressed transgenic citrus lines, and *STM* and *FIE* were also upregulated in the knockout line (Figure 3k). Compared with overexpressed transgenic citrus lines, the development of leaves shows more serious growth inhibition. In addition, multiple meristem growth points appeared in the *CiMADS43* knockout line (Figure 3f,g). Notably, the apical meristem and leaf development of the two knockout lines were inhibited compared with the control at the stage of genetic transformation. Finally, they could not develop into healthy plants and died prematurely after rooting and transplanting because the growth was severely inhibited.

### 2.4. CiAGL9 can Interact with CiMADS43 Proteins

**Figure 4:**
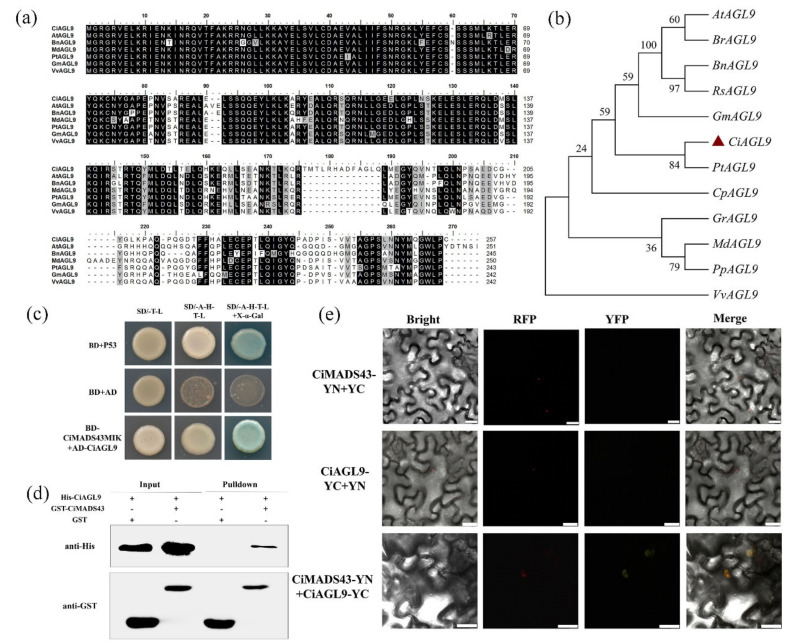
The interaction between CiAGL9 and CiMADS43 protein. (**a**) Multiple sequence alignment of CiAGL9 protein and its homolog proteins. CiAGL9, *Citrus sinensis* (NP_001306995.1). AtAGL9, *Arabidopsis* (AAC00586.1). BnAGL9, *Brassica napus* (XP_022545908.1). MdAGL9, *Malus domestica* (XP_028952174.1). PtAGL9, *Populus trichocarpa* (XP_024453001.1). GmAGL9, *Glycine max* (NP_001242742.2). VvAGL9, *Vitis vinifera* (NP_001268114.1). (**b**) Phylogenetic analysis of AGL9 proteins of citrus and other plants. The protein ID of all AGL9 proteins were listed in Table S1. (**c**) Y2H assays between CiMADS43MIK and CiAGL9 proteins. (**d**) Pull-down assay of the interaction between CiMADS43 and CiAGL9. (**e**) Bimolecular fluorescence complementation (BiFC) analysis of protein interactions between CiMADS43 and CiAGL9 in tobacco leaf epidermis cells. CiMADS43-YN + CiAGL9-YC, coexpression of 35S::CiMADS43-nYFP and 35S::CiAGL9-cYFP. CiMADS43-YN + YC and CiAGL9-YC + YN were used as the negative control. Scale bar = 25 µM.

CiMADS43 is a typical MIKC type MADS-box protein, which contains MADS domains related to dimer formation, and may form heterodimers with other proteins [4,59]. Therefore, yeast two-hybrid (Y2H) screening experiment was performed in this study. Because the full-length *CiMADS43* has a self-activation activity by the yeast system analysis, we used CiMADS43MIK that lacked the *C*-terminal 80 amino acids as bait to identify the CiMADS43 interacting proteins. A total of 15 proteins were obtained (Table S2). Among these proteins, we were interested in a MADS-box protein (Ciclev10032507m). Multiple sequence alignment (Figure 4a) and phylogenetic tree analysis found that it was a SEPALLATA (SEP) sub-family MADS-box protein which highly homologous with *Populus trichocarpa* AGL9 and named CiAGL9 (Figure 4b).

To further validate CiAGL9 interact with CiMADS43, a series of experiments were performed. Firstly, the full-length CiAGL9 was fused with GAL4-AD, and co-expressed with BD-CiMADS43MIK fusion construct in yeast cells. As expected, CiAGL9 interacted with CiMADS43 in yeast cells (Figure 4c). Secondly, the interaction of CiAGL9 with CiMADS43 in vitro was also verified by pull-down assay (Figure 4d). Finally, bimolecular fluorescence complementation (BiFC) was also carried out in tobacco. CiMADS43 was fused with the *N*-terminal of yellow fluorescent protein (YFP), and CiAGL9 was fused with the *C*-terminal of YFP. The fusion constructs were then transiently co-expressed in tobacco leaves. Yellow fluorescence was exclusively detected in the nucleus, while co-expression with an empty vector did not show any discernible YFP fluorescence (Figure 4e). These results indicated that there was an interaction between CiMADS43 and CiAGL9.

### 2.5. Expression Patterns and Promoter Analysis

**Figure 5:**
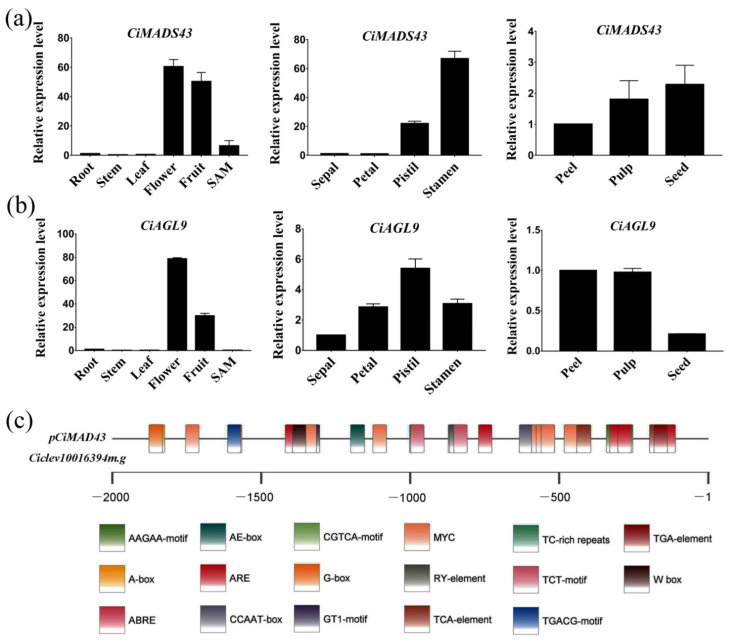
Expression patterns and promoter *cis*-elements analysis. (**a**,**b**) qRT-PCR analysis of *CiMADS43* and *CiAGL9* in different tissues. (**c**) The 2.0 kb *CiMADS43* promoter was analyzed by the PlantCARE software. Boxes with different colors represent different elements. The sequences and cis-elements were listed in Table S3.

To investigate the expression pattern of *CiMADS43* and *CiAGL9*, qRT-PCR was performed in sweet orange different tissues. The results showed that *CiMADS43* had relatively high expression levels in the SAM and fruit, and the highest expression level was observed in flower (Figure 5a). Similarly, *CiAGL9* was mainly expressed in flowers and fruit, with the highest expression in flower (Figure 5b). In flowers, *CiMADS43* was mainly expressed in pistil and stamen, and very low in sepal and petal (Figure 5a). *CiAGL9* was also high expressed in the pistil (Figure 5b). Among different parts of the fruit, *CiMADS43* had the highest expression level in seeds (Figure 5a), in which *CiAGL9* had the lowest expression level (Figure 5b). These results indicated that *CiMADS43* and *CiAGL9* may work together and also have their own unique functions in regulating flower and fruit development in citrus.

To further investigate the function of *CiMADS43*, approximately 2kb *CiMADS43* promoter was isolated based on the citrus genome [60]. The cis-elements of *CiMADS43* promoter were analyzed by PlantCARE software [61]. The results showed that the promoter contains many typical binding elements such as A-box (CCGTCC), G-box (TACGTG), and W-box (TTGACC). We also found many elements or motifs related to hormones IAA (indole-3-acetic acid), JA (jasmonic acid), ABA (abscisic acid), such as TGA-element (AACGAC), TCA-element (CCATCTTTTT/TCAGAAGAGG), ABRE element (ACGTG), TGACG-motif, and CGTCA-motif. In addition, there are some light responsive element GT1-motif (GGTTAA), TCT-motif (TCTTAC), and stress response-related elements ARE (AAACCA), and TC-rich repeats (GTTTTCTTAC). These results suggest that *CiMADS43* may have multiple functions during citrus development (Figure 5c and Table S3).

### 2.6. CiAGL9 Can Directly Bind to the Promoter of CiMADS43

**Figure 6:**
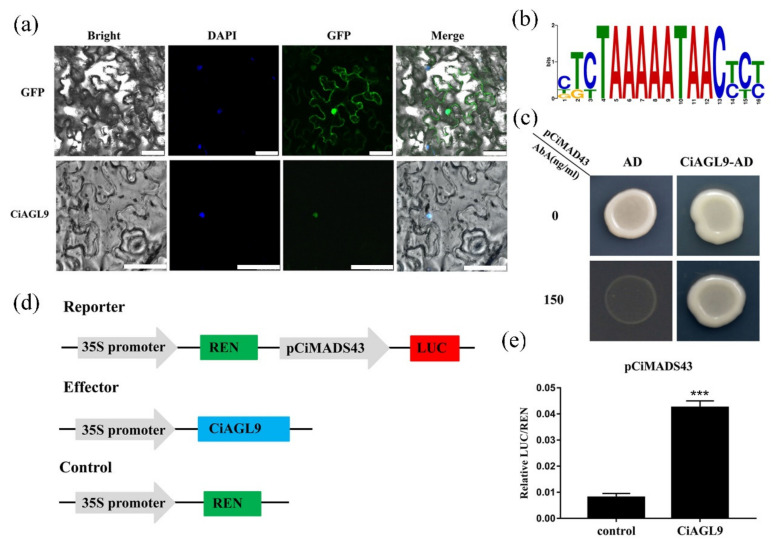
CiAGL9 binds to the CiMADS43 promoter. (**a**) Subcellular localization of CiAGL9 in tobacco leaves. GFP fused to the *C*-terminal region of CiAGL9, and the fusion protein was driven by 35S promoter. 4′,6-diamidino-2-phenylindole (DAPI) staining showed the location of the nucleus and 35S::GFP was used as the positive control. Scale bar = 25 µM. (**b**) Sequence of MADS-box protein binding site. (**c**) The interaction between the CiMADS43 promoter and CiAGL9 by yeast one-hybrid assay. pCiMADS43: 482 bp *CiMADS43* promoter sequence was cloned into pAbAi vector. AD: pGADT7 empty vector. CiAGL9-AD: CiAGL9 fused with GAL4-AD. (**d**) Schematic diagrams of vectors used for the dual-luciferase (LUC) assay. (**e**) CiAGL9 activated the expression of CiMADS43. LUC reporter system was used in tobacco leaves. The relative LUC/REN were measured after 2 days of *Agrobacterium* infiltration. Statistically significant is marked with asterisk(s) (*** *p* < 0.001, Student’s *t*-test).

To identify the regulatory factors upstream of *CiMADS43*, the 2.0 kb promoter of *CiMADS43* was divided into three fragments. The certain elements which may response to flowering time or leaf development are concentrated in the 1.1 to 1.6 kb promoter fragments (Figure 5c). Therefore, a 482 bp (from −1188 to −1670) promoter fragment was used as baits to screen yeast one-hybrid (Y1H) library. Three typical TFs were found including an ERF type transcription factor (*ERF13*, Ciclev10022025m), a bHLH type transcription factor (*UNE10*, Ciclev10020053m), and a MADS-box type TF (Ciclev10032507m). Interestingly, sequence alignment analysis found that Ciclev10032507m was exactly the same as *CiAGL9*. Therefore, we chose *CiAGL9* as an candidate protein for further analysis. Subcellular localization analysis showed that *CiAGL9* was also located in the nucleus similar to *CiMADS43* (Figure 6a). In addition, there is a GArG-box like MADS-box gene binding site in the *CiMADS43* promoter fragment (Figure 6b). Therefore, *CiAGL9* was fused with GAL4-AD and transformed into Y1H-gold yeast containing pCiMADS43-AbAi. As expected, the fused Y1H-gold cells were grown well on SD/-Leu media with or without 150 ng/mL aureobasidin A (AbA) selection, but the control AD were not grown with 150 ng/mL AbA selection. These results indicated CiAGL9 can bind to the promoter of CiMADS43 in yeast cells (Figure 6c).

To further confirm the interaction of *CiAGL9* with *CiMADS43* promoter, a dual-luciferase (LUC) reporter system was used. *CiAGL9* was cloned into the pGreenII 62-SK vector to generate CiAGL9-62SK, and the 482 bp promoter sequence was fused to LUC of the pGreenII-0800 vector to generate ProCiMADS43::LUC (Figure 6d). CiAGL9-62SK coinfiltrated with ProCiMADS43::LUC into tobacco leaves for expression. As a result, the coexpression of CiAGL9-62SK and ProCiMADS43::LUC showed stronger LUC fluorescence than the control (Figure 6e). This result suggested that *CiAGL9* bind to the *CiMADS43* promoter and activates its expression.

### 2.7. CiAGL9 Also Participate in the Regulation of Flowering Time and Leaf Development

**Figure 7:**
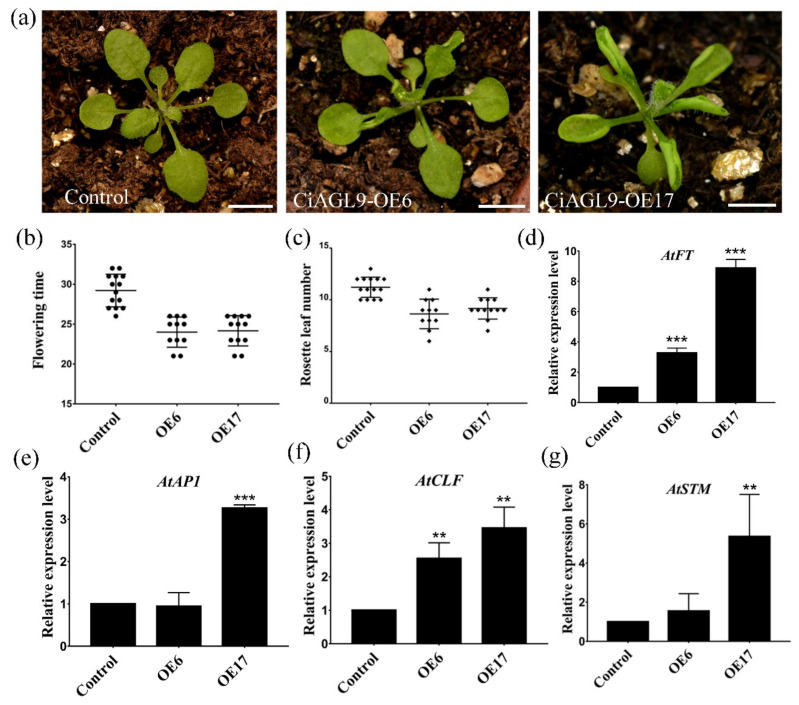
Ectopic expression of *CiAGL9* causes slightly early flowering and leaf curling in transgenic *Arabidopsis*. (**a**) The phenotype of *CiAGL9* transgenic *Arabidopsis*. Two transgenic lines (CiAGL9-OE6 and CiAGL9-OE17) were selected as representatives of 17 transgenic lines for evaluating *CiAGL9* function. Scale bar = 0.5 cm. (**b**) Statistical analysis of flowering days of the transgenic *Arabidopsis* and the control. (**c**) Statistical analysis of rosette leaves number of the transgenic *Arabidopsis* and the control at flowering stage. (**d**,**e**) qRT-PCR analysis of *Arabidopsis* flowering-related genes (*AtFT* and *AtAP1*). (**f**,**g**) qRT-PCR analysis of *Arabidopsis* leaf development related genes (*CLF* and *STM*). Statistically significant was marked with asterisk(s) (** *p* < 0.01, and *** *p* < 0.001, Student’s *t*-test).

*CiAGL9* not only interacted with *CiMADS43* protein but also bind to the *CiMADS43* promoter. Thus, we speculated that *CiAGL9* and *CiMADS43* may work cooperatively to regulate citrus flowering or leaf development. Subsequently, *CiAGL9* was also transformed into wild type *Arabidopsis*. A total of 17 transgenic lines were obtained, and most of these transgenic lines showed similar phenotypes. The leaves of transgenic plants also appeared slightly curled. The flowering time was earlier than in the control (Figure 7a). Two transgenic lines (OE6 and OE17) were selected as representatives for statistical analysis of flowering time. The average days of transgenic lines to flowering ranged from 21 to 26 days, while that of the control was 29.2 days (Figure 7b). The average number of rosette leaves ranged from 6 to 11 at flowering stage and was 11.2 in the control (Figure 7c). *AtFT* and *AtAP1* was upregulated in transgenic plants compared with the control (Figure 7d,e). Meanwhile, the genes related to leaf development were also investigated, and the results showed that *AtCLF* and *AtSTM* were also upregulated compared with the control (Figure 7f,g). These results suggested that *CiAGL9* might promote flowering and participate in the regulation of leaf development in citrus.

### 2.8. The Expression Analysis of CiMADS43 and CiAGL9 during Citrus Buds and Leaves Development

**Figure 8:**
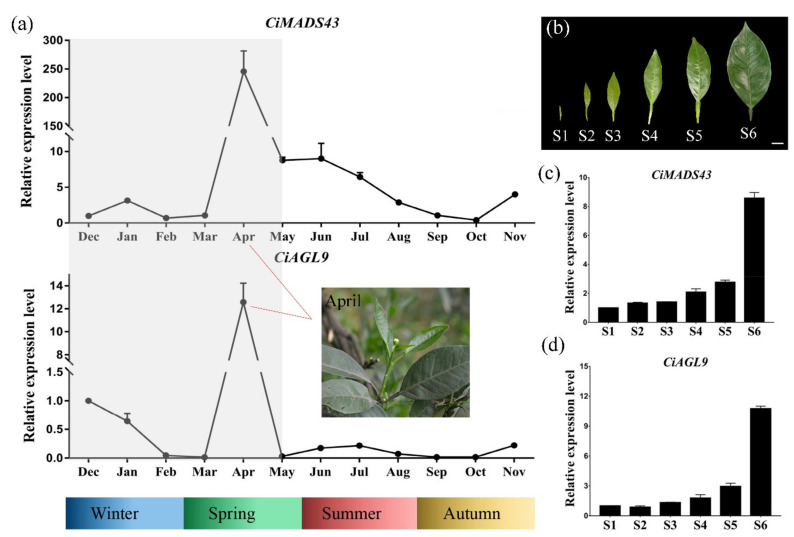
The expression patterns of *CiMADS43* and *CiAGL9* during buds and leaves development in citrus. (**a**) The seasonal expression changes of *CiMADS43* and *CiAGL9* in sweet orange buds. April is the flowering period of sweet orange. (**b**) The development process of sweet orange leaves was divided into six periods (S1–S6) from small to large. Scale bar = 1 cm. (**c**,**d**) qRT-PCR of *CiMADS43* and *CiAGL9* in the six periods of sweet orange leaf development.

*CiMADS43* and *CiAGL9* promoted early flowering of *Arabidopsis*. Therefore, to investigate whether these two genes were involved in the seasonal periodicity of flowering in citrus, their expression levels were investigated in sweet orange. The buds of sweet orange were collected every month of the year. The results indicated that the expression patterns of *CiMADS43* and *CiAGL9* were similar, and their expression levels relatively high during the months of flower bud differentiation stage (October, November, December), and reached the highest levels in April when citrus flowered, and then decreased after flowering (Figure 8a). This finding suggested that *CiMADS43* and *CiAGL9* may be involved in the seasonal periodicity of flowering and is related to citrus flowering.

To further investigate whether these two genes also participate in the development of citrus leaves, their expression levels were investigated at different developmental stages of sweet orange leaves. In this study, the development of citrus leaves was divided into six stages (Figure 8b). The results showed that the expression levels of *CiMADS43* and *CiAGL9* gradually increased with the growth of leaves, and reached the highest level at the mature stage (Figure 8c,d). These results further suggested that *CiMADS43* and *CiAGL9* may be also involved in the regulation of citrus leaf development.

## 3. Discussion

Bsister protein forms a branch of MADS-box genes that originated 300 million years ago [43]. So far, 78 Bsister genes have been identified from 51 species of Gymnosperm and Angiosperm plants [62]. Bsister genes are divided into two types: ABS-like and GOA-like [62]. Phylogenetic analysis showed that citrus has two Bsister genes, one belongs to GOA-like type and mainly expressed in flowers and seeds [62]. The other one belongs to the ABS-like type mainly expressed in seeds [62]. The expression of most Bsister genes is limited to reproductive organs, mainly the epidermis of ovules [54]. In the present study, *CiMADS43* was a GOA-like Bsister protein and mainly expressed in the flower and fruit of citrus similar to other dicotyledonous plants. In different parts of flowers and fruit, *CiMAD43* was mainly expressed in sepals, petals, and seeds. This results indicated that the Bsister gene was relatively conservative in flowers and seeds among different plant species. However, we found that *CiMADS43* also had a high expression level in SAM, which was not reported in other species. We speculated that *CiMADS43* has retained the function of the original Bsister genes and also may obtain new functions in the process of genetic evolution.

To date, Bsister genes have been functionally characterized in *A. thaliana* [28]. *AtABS* was the first Bsister gene to be functionally characterized. The inner epidermal layer of its mutant (*tt16-1*) becomes thinner and the seed develop into a straw-colored [47,63]. However, *GOA* has a non-redundant function for *ABS* in the regulation of ovule coat development and fruit longitudinal growth, and overexpression of *GOA* results in early flowering, curly-leaf that is extremely reduced in size [51]. The double mutant (*goa/tt16*) showed that they played an additive role in controlling seed coat development [28]. Our results demonstrated that *CiMADS43* has similar functions with *A. thaliana* *GOA*. Overexpressed *CiMADS43* also showed a curly-leaf phenotype in citrus. These results indicated that the function in regulating leaf cell development of GOA-like Bsister genes was relatively conserved between *A. thaliana* and citrus. Interestingly, the multiple lateral meristem appeared in citrus knockout lines, which has never been reported in Bsister gene mutants of other plants. Multiple meristems may indicate enlarged apical meristem or enhanced vegetative bud (axillary meristem) growth. This may be related to the specific expression of *CiMADS43* in citrus SAM. Therefore, the specific expression of *CiMADS43* may be involved in the regulation of the SAM development, and it was likely to maintain the apical dominance. When *CiMADS43* was knocked out, the inhibition of lateral meristems was lifted and multiple growth points were formed. It may be that *CiMADS43* gained a new function during the evolution of the Bsister gene of woody plants.

MADS-box gene usually forms homo- or heterodimers to regulates its downstream target genes [4,64]. Protein interaction analyses showed that *Arabidopsis* ABS was mediated by SEP3 to form heterodimers and tetramers with several MADS proteins, such as SEEDSTICK (STK), SHP1, and SHP2, and genetic interactions with SHP1 and SHP2 in a partial antagonistic manner to involve in the development of seeds endodermis and endosperm [63]. This interaction is very conservative in the ABS protein and almost all ABS-like proteins were able to form heterodimers with AP1, SEP1, SEP3, and SEP4 in *Arabidopsis* [4,64]. However, only one interaction partner AGL16 was identified for GOA-like protein, and the GOA orthologs of *Capsella rubella* and *Lepidium campestre* were unable to form heterodimers with *A. thaliana* AGL16 protein [64]. In this study, we found that CiAGL9 can interact with CiMADS43. Overexpression of *CiAGL9* in *Arabidopsis* also resulted in early flowering and curly-leaf phenotype, similar to that of *CiMADS43*. These results indicates that Bsister MADS-box protein may form heterodimers with other MADS-box proteins to regulate its downstream target genes. A previous study revealed that MADS-box gene family play pivotal regulatory roles in both the vernalization- and photoperiod-regulated flowering pathways [65]. In citrus, day length or photoperiod changes have little influence on floral induction [66]. Adult citrus trees in subtropical regions are induced to flower by seasonal exposure to low temperatures under natural conditions [67,68,69]. In this study, the accumulation of *CiAGL9* and *CiMADS43* was induced by seasonal low temperature. These results indicated an association between the increase in the expression of *CiAGL9* and *CiMADS43* and floral induction by seasonal low temperature. In addition, *AP1* and *LFY* were significantly upregulated in *CiAGL9* and *CiMAD43* transgenic *Arabidopsis*. Therefore, we speculate that CiAGL9 and CiMAD43 may form a complex, and then bind to the promoter of *CiAP1* and/or *CiLFY* to regulate the low temperature-induced citrus flowering.

Interestingly, yeast one-hybrid assay found that *CiAGL9* also bind to *CiMADS43* promoter and activated its expression. Therefore, there may be a feedback mechanism between *CiAGL9* and *CiMADS43*, which is not reported in the Bsister gene. Recently, this mechanism has also been reported in other species [70]. For example, the NAC transcription factor (*MaNAC2*) form a feedback regulation mechanism with a C3HC4-type RING E3 ligase (MaXB3) to regulate ethylene biosynthesis in the process of banana ripening [71]. MaXB3 interacts with MaNAC2 and destabilizes it. Meanwhile, *MaNAC2* represses *MaXB3* by binding to its promoter, resulting in a feedback regulatory mechanism that maintains a balance of *MaNAC2* levels. Similarly, *FAR-RED ELONGATED HYPOCOTYL3* (*FHY3*) and *FAR1* integrate light and strigolactone (SL) to regulate branching through a feedback regulatory mechanism in *Arabidopsis*. FHY3 and FAR1 are essential for phytochrome A-mediated light signaling. SMXL6 and SMXL7 are two key repressors of the SL signaling pathway. They form polymer complexes and directly interact with SPL9 and SPL15 to suppress their transcriptional activation of BRC1. Meanwhile, FHY3 and FAR1 upregulate the expression levels of SMXL6 and SMXL7 by binding to their promoter and thus promote branching [72]. However, the precise regulatory function between *CiAGL9* and *CiMADS43*, especially within the early flowering and leaf development, remains largely unknown and additional target genes are still to be identified.

In conclusion, *CiMADS43* and *CiAGL9* were working together in regulating flowering and leaf development in citrus. *CiAGL9* might regulate *CiMADS43* in two different ways. One is transcriptional regulation that *CiAGL9* binds to *CiMADS43* promoter and activates its expression. The other is post-transcriptional regulation that CiAGL9 forms heterodimers with CiMADS43, and then combine with the downstream genes which relate to flowering and leaf development and thus promote their expression. These findings will facilitate our understanding of the evolution and molecular mechanisms of Bsister genes in higher plants.

## 4. Materials and Methods

### 4.1. Plant Material

Sweet orange (*Citrus sinensis* L. Osbeck) and lemon (*Citrus limon*) were collected in the experiment fields of the National Citrus Breeding Center at Huazhong Agricultural University. Bud samples from sweet orange were collected at 9–11 a.m. on sunny days in the middle of each month. Meanwhile, healthy leaves were also collected at different developmental stages. To investigate the spatial expression of flowering related genes, sweet orange various tissues were also collected including root from adult trees, stem from spring shoots, healthy mature leaves, flowers at full bloom, and fruits (ripe) were collected from adult sweet orange trees. Fresh samples were collected from three groups of trees and were frozen immediately in liquid nitrogen, and then stored at −80 °C until use. In addition, lemon seeds were also collected for *CiMADS43* transformation.

### 4.2. Cloning and Sequence Analysis of CiMADS43

Total RNA was extracted using RNA simple Total RNA Kit (Tiangen Biotech, Beijing, China) according to the manufacturer’s instructions. DNA was extracted using the cetyltriethylammnonium bromide (CTAB) method [73]. First-strand cDNA was synthesized using the PrimeScript™ II 1st Strand cDNA Synthesis Kit (Takara, Japan) following the manufacturer’s instructions. The cDNA and DNA from citrus leaves were used as templates for PCR cloning. All the primers were listed in Table S4. MADS43 and AGL9 protein sequences from citrus and other plants were obtained from the National Coalition Building Institute (NCBI) and listed in Table S1. Multiple sequence alignment was carried out using ClustalW [74]. The phylogenetic tree was constructed using the neighbor-joining method by MEGA7 software [75]. To assess the promoter cis-elements of *CiMADS43*, approximately 2.0 kb of promoter fragment from the start codon of *CiMADS43* was isolated based on citrus genome [60]. PlantCARE was used for cis-element investigation [61].

### 4.3. Subcellular Localization Analysis

To investigate the subcellular localization of *CiMADS43* and *CiAGL9*, their coding sequences (CDS) without the stop codon were amplified and cloned into pBI121 vector containing the GFP reporter gene under the control of the CaMV35S promoter. The fusion constructs and control vector were integrated into *Agrobacterium tumefaciens* GV3101. Tobacco leaves were infected with GV3101 carrying either the fusion constructs or the control vector as the previously described method [76]. Red fluorescent protein (RFP) marker was used to locate the nuclei by cotransfecting. The infiltrated plants were grown for one day in the dark and one day in light prior to fluorescence signal detection using a laser scanning confocal microscope (TCS-SP8, Leica, Germany).

### 4.4. Transactivation Assay

For transactivation activity assay of *CiMADS43*, its full-length CDS was amplified using PCR and inserted into the pGBKT7 vector to fuse with a GAL4-BD. Meanwhile, three truncated sequences of *CiMADS43* were also constructed. The recombinant plasmid BD-CiMADS43 (1–747 bp), BD-CiMADS43MIK (1–504 bp), BD-CiMADS43MI (1–255 bp), BD-CiMADS43KC (256–747 bp), and empty pGBKT7 vector were separately transformed into the yeast AH109 strain. The transformed yeasts were growth on SD media lacking tryptophan (SD/-Trp) incubated at 30 °C for 3 days. Subsequently, the positive clones were transferred to SD/-Trp media, SD/-Trp-His media, and SD/-Trp-His media supplemented with x-gal. The transactivation activity of the transformants was determined based on the growth status (blue/white). If the positive clones can grow in all media and turn blue in SD/-Trp-His media supplemented with x-gal, but the control only grows in SD/-Trp, the positive clones have transcriptional activity.

### 4.5. Quantitative Real-Time Polymerase Chain Reaction (qRT-PCR)

For qRT-PCR, total RNA was extracted using RNA simple Total RNA Kit (Tiangen Biotech, Beijing, China) according to the manufacturer’s instructions. First-strand cDNA was synthesized using the PrimeScript™ II 1st Strand cDNA Synthesis Kit (Takara, Japan) following the manufacturer’s instructions. Primer sequences used for qRT-PCR were listed in Table S4. The qRT-PCR mixtures (10 µL total volumes) contained 5 µL SYBR Green (SuperReal PreMix Plus, Tiangen), 0.2 µL of each primer (10 µM), 0.4 µL cDNA, and 4.2 µL RNase-free water. The reactions were carried out using the Power SYBR Green PCR Master Mix (Applied Biosystems) in an ABI 7500 Sequence Detection System using the following thermocycling conditions: initial enzyme activation for 5 min at 95 °C, followed by 40 cycles of denaturation for 10 s at 95 °C, annealing for 20 s at 60 °C, and extension for 30 s at 72 °C, with a melting curve analysis performed upon completion of the reaction. Each sample was run in triplicate, with three biological replicates. The data were normalized to *actin* transcript levels to minimize variation in cDNA template levels. Results was calculated by using the 2^−ΔΔCt^ method and was presented as mean relative transcript levels standard error of three biological replicates.

### 4.6. Vector Construction and Gene Transformation

For plant transformation, the CDS of *CiMADS43* and *CiAGL9* was amplified with a specific forward primer designed to introduce a *BamH* I restriction site and a reverse primer designed to introduce an *EcoR* I restriction site to allow subcloning (Table S4). After PCR and digestion, the *CiMADS43* and *CiAGL9* fragments were inserted into the pBI121 vector. The recombined vector was transferred into *Agrobacterium tumefaciens* GV3101 strain using the freeze-thaw method. *Arabidopsis* was transformed by floral-dip method [77]. *Agrobacterium*-mediated lemon (Femninello) transformation of stem segments was performed as previously described [78,79]. The positive lines were screened by PCR amplification of genomic DNA. The mRNA abundance of *CiMADS43* in transgenic lemon lines was detected by qRT-PCR.

### 4.7. CRISPR/Cas9 Vector Construction and Genotyping Analysis

The P201N-Cas9 vector was used to construct a plasmid vector expressing Cas9 and sgRNA simultaneously in lemon. The Cas9 gene was codon-optimized for dicotyledons and placed downstream of the StUbi-3 promoter together with customized sgRNA driven by the MtU6 promoter. We designed the sgRNAs using the web-based tool CRISPR-P2.0 [80] and used CRISPR web tool to predict the off-target sites [81]. The primers used for detection vector construction, sequencing and *CiMADS43* genotyping are all displayed in Table S4.

### 4.8. Yeast Two-Hybrid Assay

Total RNA from different tissues of sweet orange was used to construct a yeast library, and roughly equal numbers of plant tissues were pooled. Yeast two-hybrid library was constructed using the Matchmaker^®^ Gold Yeast Two-Hybrid System following the manufacturer’s kit instructions (Takara Bio, Beijing, China). The CDS region of *CiMADS43MIK* and *CiAGL9* was inserted into pGBKT7 and pGADT7 vectors to generate pGBKT7-CiMADS43MIK and pGADT7-CiAGL9, respectively. These recombined constructs were cotransformed into yeast AH109 strain following the manufacturer’s manual (Takara Bio, Beijing, China). The pGADT7 and pGBKT7 vectors were used as negative controls. The transformants were selected by growth on SD/−Leu/−Trp medium at 30 °C for 3–4 days. The interactions were tested by growth on SD/−Ade/−His/−Leu/−Trp medium with or without X-α-Gal. All experiments were repeated three times.

### 4.9. Bimolecular Fluorescence Complementation (BiFC)

To construct the fusion vector pSPYNE-CiMADS43 and pSPYCE-CiAGL9, their CDSs were cloned into pSPYNE vector and pSPYCE vector, respectively. The fusion vector was introduced into *Agrobacterium tumefaciens* GV3101. The resuscitated pSPYNE-CiMADS43 and pSPYCE-CiAGL9 *Agrobacterium* were separately mixed with the nuclear marker. The final OD_600_ of each *Agrobacterium* tumefaciens suspension was set to 0.8, and 50 mM acetosyringone was added, and then transfected into 5-week-old tobacco. After incubating in the dark for one day and incubating under the light for one day, the fluorescence of YFP can be observed with a laser confocal microscope (TCS-SP8, Leica, Munich, Germany).

### 4.10. Pull-Down Assay

The CDS of *CiAGL9* was cloned into the pET32a vector to generate CiAGL9-His fusion protein, and the CDS of *CiMADS43* was cloned into the pGEX-6p-1 vector to generate the CiMADS43-GST fusion protein. The constructs of CiAGL9-His and CiMADS43-GST were transformed into *Escherichia coli* BL21 (DE3) strain and induction with 1 mM Isopropyl β-d-thiogalactoside (IPTG) during 16 h at 16 °C. All the cells were harvested by centrifugation at 4000× *g* for 20 min at 4 °C. Total proteins were extracted with the buffer (pH 7.5) containing 0.7 M sucrose, 0.1 M KCl, 0.5 M Tris-HCl, 50 mM EDTA, 2% (*v*/*v*) β-mercaptoethanol, and 100 mM phenylmethylsulfonyl fluoride (PMSF). The protein concentration was determined using the BCA reagent (Aidlab) with bovine serum albumin as a standard. CiAGL9-His were incubated with CiMADS43-GST or GST in binding buffer (400 mM NaCl, 50 mM Tris-HCl, 1 mM PMSF, and 2% (*v*/*v*) β-mercaptoethanol, pH 8.0) at 4 °C overnight. Glutathione Beads (Smart-Lifesciences, SA008K, Changzhou, China) were washed five times with the pull-down buffer. The bound proteins were released by adding 2× loading buffer and boiled for 5 min at 95 °C, then resolved by sodium dodecyl sulfate polyacrylamide gel electrophoresis (SDS-PAGE) and detected by the anti-His (TransGen Biotech, HT501-01) and anti-GST antibodies (TransGen Biotech, HT601-01, Nanjing, China), respectively.

### 4.11. Yeast One-Hybrid Assay

The *CiMADS43* promoter fragment (from −1 to −1670) was amplified, and potential cis-elements were predicted by PlantCARE software [61]. The core region (from −1188 to −1670) of *CiMADS43* promoter was inserted into the pAbAi vector with the ClonExpress One Step Cloning Kit (Vazyme Biotech Co., Ltd., Nanjing, China). The bait vector pCiMADS43-AbAi was transformed into the yeast Y1H-Gold strain and test self-activation with SD/-Ura medium under different concentrions of AbA. The yeast one-hybrid library was constructed using the Matchmaker Gold Yeast One-Hybrid Library Screening System (Clontech, Palo Alto, CA, USA) in accordance with the manufacturer’s protocol (protocol #PT4087-1). Total RNA from different tissues of sweet orange was prepared as described previously for the yeast two-hybrid assay. Prepare Y1H-gold yeast competence containing pCiMADS43-AbAi, and then used as baits to screen against a library of citrus TFs with SD/-Leu+AbA medium. After culturing at 30 °C for 3 days, single clones were picked for cloning detection. The preparation of yeast competence and plasmid transformation refer to Matchmaker Gold Yeast One-hybrid System kit (Takara Bio, Beijing, China). Both positive (pGADT7-p53 + p53-AbAi) and negative (pGADT7 + pCiMADS43-AbAi) controls were included and handled in the same manner.

### 4.12. Dual Luciferase (LUC) Assays

The CDS of *CiAGL9* was fused into the pGreenII 62-SK vector using the CloneExpressTM II One Step Cloning Kit (Vazyme Biotech Co., Ltd., Nanjing, China) to generate an effector construct, while the *CiMADS43* promoter fragment was inserted into pGreenII 0800-LUC to generate reporters construct. The effector and reporter vectors were mixed and co-infiltrated into tobacco leaves as previously described method [76]. Tobacco leaf cells co-transformed with the reporters and the empty vector pGreenII 62-SK were used as control. A Dual-Luciferase Reporter Assay System (Promega, Madison, WI, USA, Cat.#E1910) with an Infinite200 Pro microplate reader (Tecan) was used to measure the ratio of luminescence of firefly LUC to Renilla LUC according to the manufacturer’s instructions.

## Supplementary Materials

The following are available online at https://www.mdpi.com/article/10.3390/ijms22105205/s1, Figure S1. The rosette leaves of CiMADS43 transgenic Arabidopsis with varying degrees of curling. Figure S2. Expression analysis of CiMADS43 in overexpressed transgenic lemon and wild type (WT). Figure S3. CiMADS43 gene and protein sequences of wild-type and mutation types at target sites. (a) CiMADS43 gene sequences at target sites. Blue, 20 bp gRNA target sites. red, PAM region. Dashes, deletions. (b) CiMADS43 protein sequences of MADS-domain. Red, High consensus. Blue, Low consensus. Dashes, deletions. Table S1. Proteins used for phylogenetic analysis. Table S2. The proteins obtained by Y2H screening. Table S3. Analysis of CiMADS43 promoter sequences. Table S4. The primers used in this study.
