# Supplementary material for: A MADS-Box Gene CiMADS43 Is Involved in Citrus Flowering and Leaf Development through Interaction with CiAGL9
Source: Int J Mol Sci. 2021 May 14;22(10):5205. doi: 10.3390/ijms22105205 (PMC8156179; doi:10.3390/ijms22105205)
Supplement: Supplementary file 1 [file ijms-22-05205-s001.zip › Supporting Information Figures S1-S3.pdf]

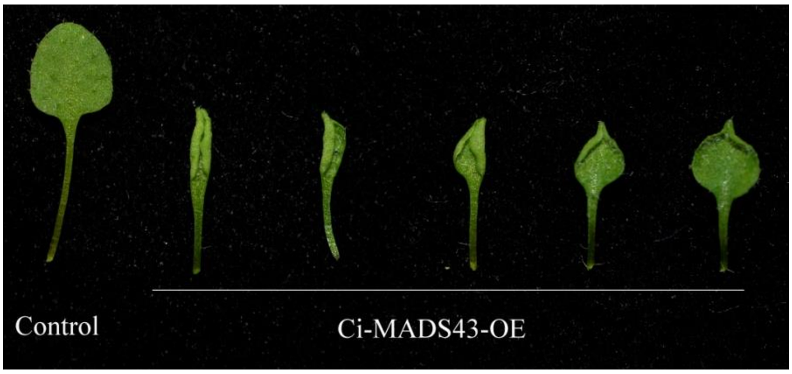

Figure S1. The rosette leaves of *CiMADS43* transgenic *Arabidopsis* with varying degrees of curling.

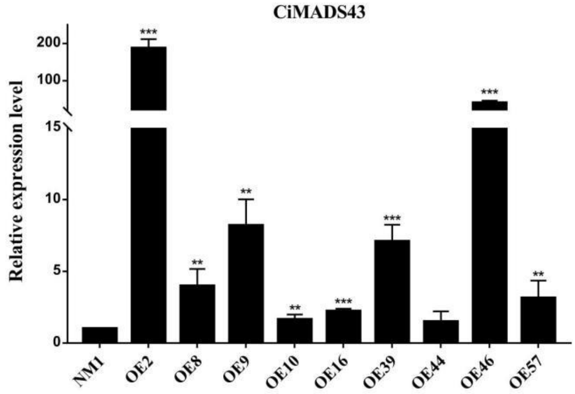

Figure S2. Expression analysis of *CiMADS43* gene in overexpressed transgenic lemons and wild type (WT) .

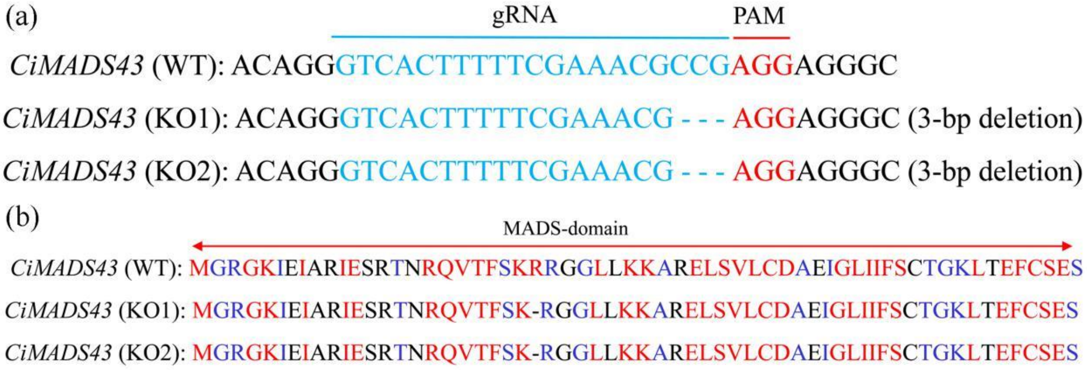

Figure S3. CiMADS43 gene and protein sequences of wild-type and mutation types at target sites. (a) CiMADS43 gene sequences at target sites. Blue, 20 bp gRNA target sites. red, PAM region. Dashes, deletions. (b) CiMADS43 protein sequences of MADS-domain. Red, High consensus. Blue, Low consensus. Dashes, deletions.
